# Genetic Risk Score for Prediction of Coronary Heart Disease in the Korean Genome and Epidemiology Study

**DOI:** 10.31083/j.rcm2404102

**Published:** 2023-04-04

**Authors:** Hyunok Yun, Ji Eun Lim, Eun Young Lee

**Affiliations:** ^1^Department of Nursing, Catholic Kkottongnae University, 28211 Cheongju, Republic of Korea; ^2^Department of Biochemistry and Molecular Biology, School of Medicine, Kyung Hee University, 02447 Seoul, Republic of Korea

**Keywords:** coronary heart disease, genetic risk score, risk prediction

## Abstract

**Background:**

Using a genetic risk score (GRS) to predict coronary heart disease (CHD) may detect disease earlier. The current study aims to assess whether GRS is associated with CHD incidence and whether it is clinically useful for improving prediction using traditional risk factors (TRFs) as well as family history.

**Methods:**

Data from a total of 48,941 participants in the Korean Genome and Epidemiology Study were analyzed in the current study. The weighted GRS was constructed using 55 single-nucleotide polymorphisms based on published genome-wide association studies. The association of GRS with incident CHD was analyzed using Cox proportional hazard model. Discrimination and reclassification were assessed to demonstrate the clinical utility of GRS. The analyses were performed separately by sex.

**Results:**

After adjusting for family history and TRFs, GRS was significantly associated with CHD incidence in men; compared to the low GRS group, men in the high GRS group had a 2.07-fold increased risk of CHD (95% confidence interval [CI]: 1.51–2.85). In men, the combination of TRFs, family history, and GRS had better performance than TRFs alone (C statistics for TRF-only model, 0.66, 95% CI, 0.64–0.69; C statistics for combination model, 0.68, 95% CI, 0.65–0.71; category-free reclassification index, 15%). In women, however, there was no significant association between GRS and CHD and no improvement between models.

**Conclusions:**

GRS was associated with CHD incidence and contributed to a small improvement of CHD prediction in men. The potential clinical use of GRS may not outweigh the value of family history.

## 1. Introduction

Coronary heart disease (CHD) is the leading cause of premature mortality and disease burden worldwide, and early detection of individuals at high risk for CHD is important for primary prevention. CHD is a complex multifactorial disease caused by a combination of genetic, cardiometabolic, behavioral, environmental, and social risk factors [1].

Major CHD risk-assessment tools have been developed. The Framingham Risk Score of the United States [2], the Risk Score of the American College of Cardiology (ACC)/American Heart Association (AHA) [3], the Systemic Coronary Risk Evaluation (SCORE) based on a large European cohort [4], and the QRISK calibrated fit to the United Kingdom (UK) population [5] have been used to identify individuals at risk for CHD. Since some of these risk-assessment tools overestimate the risk for CHD in the Korean population [6, 7, 8], the Korean CHD risk score (KRS), which incorporates age, blood pressure (BP), total cholesterol, high-density lipoprotein (HDL) cholesterol, smoking, and diabetes mellitus (DM), was also designed [9].

The heritability of CHD has been estimated to be 40%–60% [10]. Family history has long been known as a risk factor for CHD. Genome-wide association studies (GWAS) has contributed to the discovery of significant individual genetic predispositions to CHD. During the past decade, GWAS have enabled the development of a genetic risk score (GRS) consisting of a selection of genomic variants and their associated GWAS-derived weights for CHD [11, 12, 13, 14]. The GRS suggests a strong association with CHD and can potentially play an important role in primary prevention by detecting early individuals at high risk of CHD. However, its adoption in routine clinical practice remains a matter of debate. Thus, it is unclear to what extent GRS can improve CHD risk assessment when combined with traditional risk factors (TRFs), which include cardiometabolic and behavioral risk factors. Some studies [12, 15, 16] have reported an enhanced risk stratification, while others [17, 18, 19] determined that GRS does not contribute substantially to improvement of CHD prediction accuracy.

Moreover, the vast majority of CHD prediction studies using both TRFs and GRS has been conducted on populations of European descent [20]. A lack of data from Asian populations drove the current study. Thus, this study intends to determine whether GRS is associated with onset of new CHD within the follow-up period using data from the Korean population and whether GRS is clinically useful by improving CHD prediction over validated algorithms already in use, such as traditional CHD risk-assessment tools or family history.

## 2. Method

### 2.1 Study Participants

**Fig. 1.**
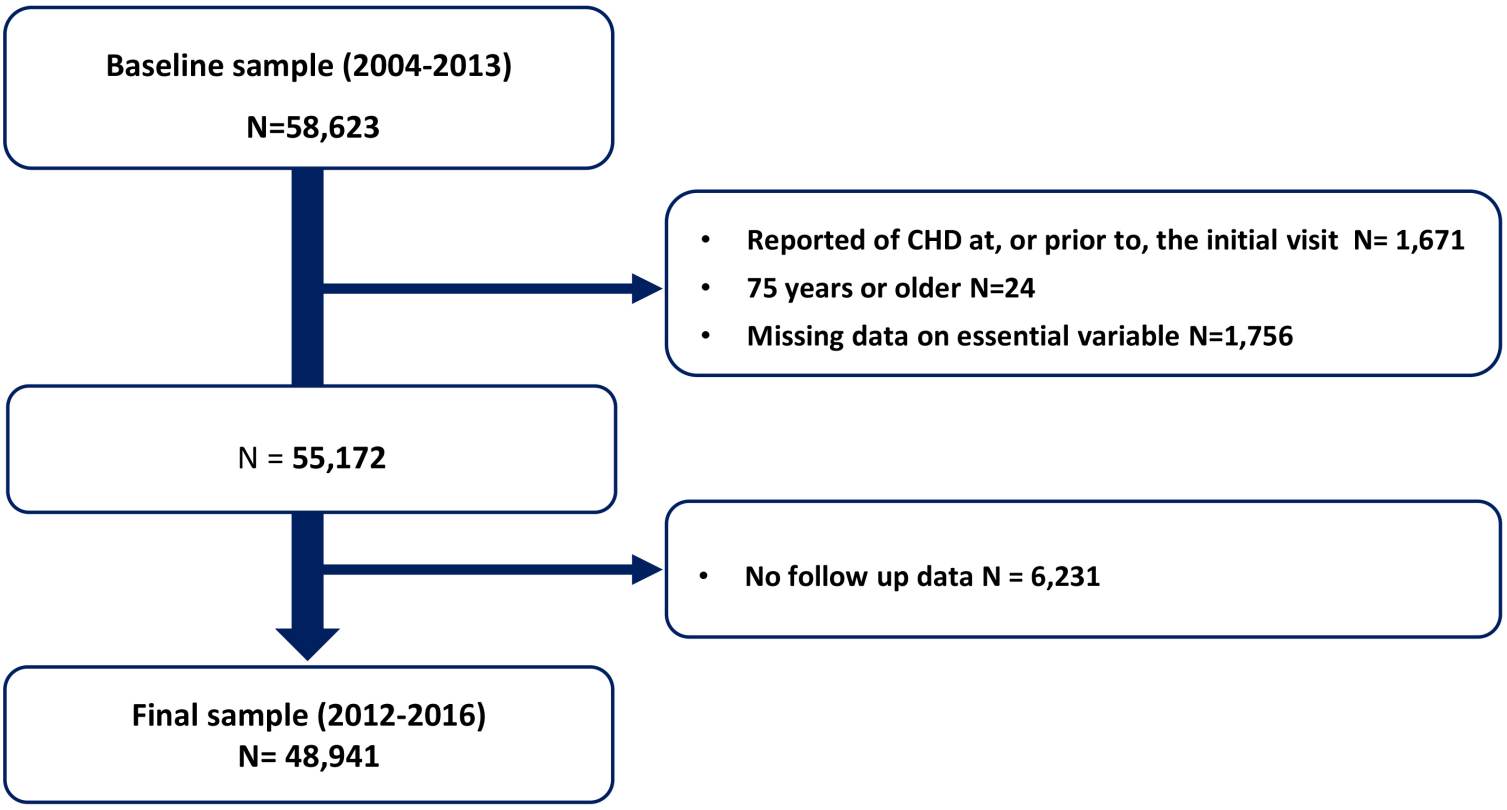
**Flow chart of selection of study participants**. CHD, coronary heart disease.

The Health Examinees (HEXA) study of the Korea Genomic Epidemiology Research (KoGES) project was established to investigate the epidemiologic characteristics, genomic characteristics, and gene–environment interactions of chronic diseases [21]. The baseline survey was performed from 2004–2013, during which 167,169 participants aged 40–69 years attended 38 health examination centers and training hospitals located in eight regions in South Korea. Among them, 58,623 participants with genomic information were included in the present study. Participants who reported CHD at baseline and those who did not participate in follow-up were excluded. Based on the same criteria as those developed in the KRS [9], participants aged ≥75 years or those without data on covariates were excluded. Based on the above criteria, a total of 48,941 participants was finally selected for study inclusion (Fig. 1).

The HEXA study protocol was approved by the Ethics Committee of the Korea National Institute of Health (KNIH) [21]. All participants provided written informed consent to participate in the study. Use of HEXA data was approved by the institutional review board of Catholic Kkottongnae University (2-7008080-A-N-01-202005-HR-002).

### 2.2 Outcome Variables

The main endpoint of this study was self-reported medical history of CHD, including angina and myocardial infarction, at follow-up examination. Subjects were classified based on answering ‘yes’ or ‘no’ to questions of diagnosis of angina or myocardial infarction by a doctor.

### 2.3 Traditional Risk Factors

Data on demographic characteristics, lifestyle factors, and medical history of study participants were obtained through individual interview using a structured questionnaire by trained experts familiar with the KoGES survey guidelines. Clinical measurements, including blood pressure, blood sugar, and lipid profiles, were obtained according to the KoGES survey guidelines. A detail explanation of the methods used to examine these indicators is available in previous publications [21, 22].

The KRS incorporated TRFs for CHD, including sex, age, BP, total cholesterol, HDL cholesterol, smoking, and DM [9]. Based on the 2018 guideline for management of hypertension by the Korean Society of Hypertension [23], BP was classified into four groups as follows; systolic BP [SBP] <120 mmHg and diastolic BP [DBP] <80 mmHg; SBP = 120–139 mmHg or DBP = 80–89 mmHg; SBP = 140–159 mmHg or DBP = 90–99 mmHg; and SBP ≥160 mmHg or DBP ≥100 mmHg. Total cholesterol (<160 mg/dL, 160–199 mg/dL, 200–239 mg/dL, 240–279 mg/dL, or ≥280 mg/dL) and HDL cholesterol (<35 mg/dL, 35–44 mg/dL, 45–49 mg/dL, 50–59 mg/dL, or ≥60 mg/dL) were grouped into five categories each. Smoking status was classified as non-smokers, former smokers, and current smokers, and DM was defined by self-reported medical history of diabetes, fasting serum glucose ≥126 mg/dL, or glycosylated hemoglobin ≥6.5% [24]. In addition, family history of CHD was obtained by self-report.

### 2.4 Genetic Risk Score Calculation

All participants were genotyped using the KoreanCHIP array designed by the Center for Genome Science of KNIH based on UK Biobank Axiom® array [25]. Single-nucleotide polymorphisms (SNPs) were imputed using IMPUTE version 2 [26] using Phase 3 of the 1000 Genomes project as a reference. Among imputed SNPs, those of low quality were filtered based on an INFO score <0.4, minor allele frequency (MAF) ≤0.01, and Hardy–Weinberg equilibrium (HWE) *p*-value ≤ 1 × ${\mathrm{10}}^{-6}$. Following SNP quality control, 7,104,351 SNPs were used for further analysis.

We constructed a coronary artery disease (CAD) GRS based on a large-scale GWAS using a Japanese population (29,319 CAD cases and 183,134 controls), which reported 57 CAD SNPs [27]. Among these, 55 SNPs were included in our imputed data. The CAD-weighted GRS was calculated as a weighted sum of risk allele counts, and the beta coefficients estimated from CAD GWAS were used as the weights of risk alleles (**Supplementary Table 1**) [27]. To calculate the GRS, we first calculated an individual’s weighted score, summing the CAD risk effects for each SNP as follows: $w\,e\,i\,g\,h\,t\,e\,d\,s\,c\,o\,r\,e=\beta_{1}\times S\,N\,P_{1}+\beta_{2}\times S\,N\,P_{2}+\cdots +\beta_{55}\times S\,N\,P_{55}$. We then generated a GRS by rescaling the weighted scores for each individual to represent the number of CAD risk alleles: $G\,R\,S=\frac{w\,e\,i\,g\,h\,t\,e\,d\,s\,c\,o\,r\,e\times n\,u\,m\,b\,e\,r\,o\,f\,a\,v\,a\,i\,l\,a\,b\,l\,e\,S\,N\,P\,s}{s\,u\,m\,o\,f\,t\,h\,e\,\beta \,c\,o\,e\,f\,f\,i\,c\,i\,e\,n\,t\,s\,o\,f\,a\,v\,a\,i\,l\,a\,b\,l\,e\,S\,N\,P\,s}$ [27].

The GRS was divided into quartiles and classified into three groups: Low-risk group (first quartile), intermediate-risk group (second and third quartiles), or high-risk group (fourth quartile).

### 2.5 Statistical Analysis

The association between GRS and time to CHD event was evaluated in a Cox proportional hazards model. Analyses were performed separately for men and women. First, we determined whether the proportional-hazards assumption was satisfied [28]. Four models were developed. Model 0 included risk factors of KRS of age, BP, total cholesterol, HDL cholesterol, smoking, and DM. Model 1 added GRS to model 0, and model 2 added family history to model 0. Model 3 included the risk factors of KRS, GRS, and family history.

To evaluate the ability of GRS to classify risks, the area under the receiver operating characteristics curve (AUC) was compared among models to assess improvement in discrimination. Even if the associations between covariates and CHD events did not reach statistical significance, relationships could be modified by exclusion and were maintained in the model for comparison with the existing predictive model. The risk categories generally established during reclassification analysis are applied over a period of 10 years; thus, they could not be directly applied to this study with its short follow-up period [29]. As such, an alternative method when the application is confusing is to use a category-free reclassification index (cNRI), which was calculated in this study [30]. Additionally, the integrated discrimination index (IDI) was calculated to evaluate model performance regardless of risk category selection [31]. Data were analyzed using R software, version 3.3.0 for Windows (The R Foundation for Statistical Computing, Vienna, Austria).

## 3. Results

Among the subjects of the HEXA cohort with genetic information, a total of 48,941 participants (32,183 women) without CHD at baseline were included in this study. Table 1 presents the characteristics of the participants. The average age at the baseline was 53.7 ± 8.0 years, and approximately 7.3% of participants had a family history of CHD. There were 650 incident cases of CHD (1.3%) during an average follow-up of 4.6 years.

**Table 1.** **Participant characteristics by sex**.

		Overall	Men	Women	*p*-value*
		(n = 48,941)	(n = 16,758)	(n = 32,183)	(t or χ^2^)
Age (years)		53.7 (± 8.0)	55.0 (± 8.4)	53.0 (± 7.6)	<0.001
SBP (mmHg)		122.3 (± 14.8)	125.7 (± 14.1)	120.6 (± 14.8)	<0.001
DBP (mmHg)		75.8 (± 9.6)	78.5 (± 9.5)	74.4 (± 9.4)	<0.001
Hypertension (mmHg)					
	SBP <120 and DBP <80	18,346 (37.5)	4446 (26.5)	13,900 (43.2)	<0.001
	SBP = 120–139 or DBP = 80–89	26,843 (54.8)	10,549 (63.0)	16,294 (50.6)	
	SBP = 140–159 or DBP = 90–99	3404 (7.0)	1587 (9.5)	1817 (5.6)	
	SBP ≥160 or DBP ≥100	348 (0.7)	176 (1.1)	172 (0.5)	
Total cholesterol (mg/dL)		197.5 (± 35.1)	192.9 (± 34.1)	199.9 (± 35.4)	
	<160	6458 (13.2)	2679 (16.0)	3779 (11.7)	<0.001
	160–199	20,359 (41.6)	7323 (43.7)	13,036 (40.5)	
	200–239	16,406 (33.5)	5278 (31.5)	11,128 (34.6)	
	240–279	4827 (9.9)	1282 (7.7)	3545 (11.0)	
	≥280	891 (1.8)	196 (1.2)	695 (2.2)	
HDL cholesterol (mg/dL)		53.5 (± 12.8)	49.1 (± 11.7)	55.8 (± 12.8)	
	<35	1911 (3.9)	1180 (7.0)	731 (2.3)	<0.001
	35–44	10,893 (22.3)	5440 (32.5)	5453 (16.9)	
	45–49	7803 (15.9)	3020 (18.0)	4783 (14.9)	
	50–59	14,273 (29.2)	4247 (25.3)	10,026 (31.2)	
	≥60	14,061 (28.7)	2871 (17.1)	11,190 (34.8)	
Smoking status					
	Non-smoker	36,039 (73.6)	4808 (28.7)	31,231 (97.0)	<0.001
	Former smoker	7649 (15.6)	7273 (43.4)	376 (1.2)	
	Current smoker	5253 (10.7)	4677 (27.9)	576 (1.8)	
DM (yes) †		4560 (9.3)	2188 (13.1)	2372 (7.4)	<0.001
Family history (yes)		3588 (7.3)	1017 (6.1)	2571 (8.0)	<0.001
Incident CHD event (yes)		650 (1.3)	336 (2.0)	314 (1.0)	<0.001

Values are presented as mean (± SD) or n (%). *Abbreviations:* CHD, coronary heart disease; DBP, diastolic blood pressure; DM, diabetes mellitus; HDL, high-density lipoprotein; SBP, systolic blood pressure. * Significance difference in proportion or mean among groups tested by Chi-square test or *t*-test, respectively. † DM was defined by self-reported medical history of diabetes, fasting serum glucose ≥126 mg/dL, or glycosylated hemoglobin ≥6.5%.

The association of GRS with CHD incidence in men and women is shown in Table 2. For men, family history and GRS were confirmed to be significant predictors. After adjusting for both TRFs and family history, the risk of CHD increased by 2.07 in the high-risk GRS group compared to the low-risk GRS group (hazard ratio [HR], 2.07, 95% CI, 1.51–2.85). For women, after adjusting for TRFs, the risk of incident CHD increased by 1.65 in those with family history of CHD compared to those without family history (HR, 1.65, 95% CI, 1.16–2.36). However, a statistically significant association between GRS and CHD incidence was not observed in women.

**Table 2.** **Associations between genetic risk score and coronary heart disease incidence by sex**.

		Men			Women		
		Model 1	Model 2	Model 3	Model 1	Model 2	Model 3
		HR (95% CI)	HR (95% CI)	HR (95% CI)	HR (95% CI)	HR (95% CI)	HR (95% CI)
GRS (ref. low risk)							
	Intermediate risk	1.40 (1.03, 1.89)		1.39 (1.02, 1.89)	1.27 (0.95, 1.68)		1.25 (0.95, 1.66)
	High risk	2.10 (1.53, 2.88)		2.07 (1.51, 2.85)	1.11 (0.80, 1.55)		1.10 (0.79, 1.54)
Family history (ref. no)							
	Yes		1.93 (1.34, 2.77)	1.88 (1.31, 2.70)		1.65 (1.16, 2.36)	1.64 (1.15, 2.34)
Concordance (SE)		0.67 (0.016)	0.67 (0.016)	0.68 (0.016)	0.71 (0.015)	0.71 (0.015)	0.71 (0.015)
Likelihood ratio test		139.6 (*p *< 0.001)	127.0 (*p *< 0.001)	149.6 (*p *< 0.001)	177.9 (*p *< 0.001)	181.6 (*p *< 0.001)	184.4 (*p *< 0.001)

Abbreviations: CI, confidence interval; GRS, genetic risk score; HR, hazard ratio; Ref., reference; SE, standard error. Model 0 included the factors of the Korean Coronary Heart Disease Risk Score (age, age*age, blood pressure, total cholesterol, high-density lipoprotein cholesterol, smoking status, and diabetes); Model 1 added GRS to model 0, model 2 added family history to model 0, and model 3 added GRS and family history to model 0.

**Fig. 2.**
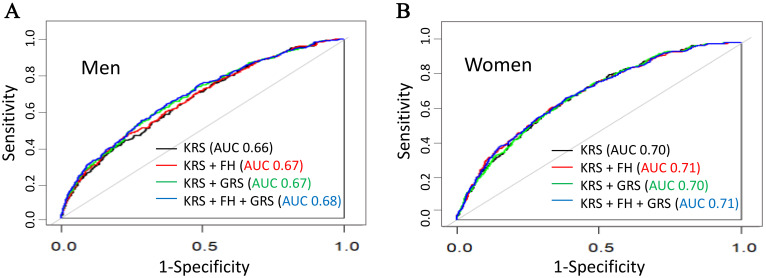
**Receiver operating characteristic curves and C statistics for models of incident coronary heart disease**. (A) Men and (B) women. *Abbreviations:* AUC, area under the receiver operating characteristic curve; FH, family history; GRS, genetic risk score; KRS, Korean coronary heart disease risk score.

The comparison of the models through discrimination and reclassification analysis is shown in Table 3 and Fig. 2. For men, the AUC value was marginally improved from 0.66 to 0.67 (*p* = 0.027) when GRS was added to the KRS. The AUC value increased significantly from 0.66 to 0.68 (*p* = 0.016) when comparing the KRS-only model with the final model including all factors. By cNRI metric, the improvement of reclassification was modest. For women, the AUC value of the KRS-only model was 0.70, which was higher than that for men; however, there was no significant improvement in discrimination when GRS and family history were added to the KRS-only model (**Supplementary Table 2, Supplementary Fig. 1,2**) .

**Table 3.** **Evaluation of genetic risk scores for prediction of coronary heart disease by sex**.

Model		Discrimination, AUC (95% CI)			Reclassification	
		Model Without GRS	Model With GRS	*p* value for difference	cNRI (95% CI)	IDI (95% CI)
Men	KRS*	0.66 (0.63, 0.69)	0.67 (0.65, 0.70)	0.027	0.15 (0.04, 0.25)	0.003 (0.001, 0.005)
	KRS + FH	0.67 (0.64, 0.69)	0.68 (0.65, 0.71)	0.036	0.15 (0.04, 0.25)	0.003 (0.001, 0.005)
Women	KRS	0.70 (0.68, 0.73)	0.70 (0.68, 0.73)	0.529	0.06 (–0.05, 0.17)	0.001 (–0.0001, 0.002)
	KRS + FH	0.71 (0.68, 0.73)	0.71 (0.68, 0.74)	0.472	0.10 (–0.01, 0.21)	0.001 (0.00002, 0.002)

Abbreviations: AUC, area under the receiver operating characteristic curve; CI, confidence interval; cNRI, category-free net reclassification Index; FH, family history; GRS, genetic risk score; IDI, integrated discrimination index; KRS, Korean coronary heart disease risk score. * The KRS was calculated by sex, age, blood pressure, total cholesterol, high-density lipoprotein cholesterol, smoking status, and diabetes.

## 4. Discussion

This study confirmed a significant association between GRS constructed with 55 SNPs and CHD incidence in Korean men after adjusting for both TRFs and family history. In men, there was marginally significant improvement with the combined model of KRS and GRS compared with the KRS-only model. However, women showed no association between GRS and CHD and no improvement between models.

Similar to our findings, Hajek *et al*. [32] reported in 2018 that a CHD GRS calculated with 46 SNPs was associated with increased risk for incident CHD among men in the Multi-Ethnic Study of Atherosclerosis cohort. The risk of CHD in white men was increased by 1.92 (95% CI, 1.19–3.11) in the highest risk GRS group compared to the lowest. However, this was not found among women. Pechlivanis *et al*. [33] likewise reported in 2020 that GRS constructed with 70 SNPs was significantly associated with CHD only in men. These findings suggest the need for further studies. The relatively small sample size and lower CHD incidence in women compared to men may have contributed to these findings, and larger studies could help to discern whether the GRS is associated with CHD incidence in women [32, 33]. Since sex chromosomes have been ignored in GWAS of CHD, there may be unidentified genetic variants for women [34]. Therefore, future studies should consider a GRS based on sex-stratified GWAS as well as that considering genetic variants of chromosome X [32, 33, 34]. Additionally, sex hormones should be considered due to their association with elevated risk for CHD events in postmenopausal women [35].

Consistent with previous studies [19, 36], adding GRS to the KRS led to a significant increase in the CHD risk stratification provided by KRS alone. However, the incremental values of GRS were modest and the significant increase was observed only in men. Elliott *et al*. [19] suggested that the incremental value of new predictors may vary depending on the discrimination potential of the existing model. In this study, the AUC values in the KRS-only model were 0.66 in men and 0.70 in women. When GRS was added to the KRS, significant improvements in discrimination ability were seen among men, which may reflect the relatively poorer performance of KRS in men compared with women [6, 9]. Although our findings showed improvement of discrimination and reclassification in the combination model of GRS and KRS, they support conclusions from previous studies that adding GRS would not yield a clinically meaningful impact to well-established comprehensive CHD risk-assessment tools such as KRS [15, 19]. However, Riveros-Mckay *et al*. [16] showed in 2021 that CAD GRS was the best-performing single risk factor in men aged <55 years. Since earlier CHD events may be more genetically determined than later events, assessment of GRS for CHD in young populations may facilitate earlier primary prevention [15, 16, 19].

Although identification of more SNPs may improve risk prediction, at least in our study, a family history of CHD compensated for this. Family history incorporates shared genetics, shared behaviors, and shared environments in families. Family history is an easily identifiable risk factor, albeit sometimes an uncertain one [37]. Tada *et al*. [38] showed that subjectively measured family history of CHD and objectively measured GRS were not redundant. Our findings also indicate that both family history and GRS should be assessed to reveal genetic predisposition.

This study has several limitations. First, it enrolled a middle-aged Korean population, so its generalizability to other ethnicities or age groups is uncertain. Second, KRS was developed to predict the 10-year risk, while the average follow-up period in this study was 4.6 years. Third, the current study did not include hard endpoints of CHD, such as a sudden cardiac death, which were included in outcome variables when developing the KRS [9]. Fourth, since the predictive power of the GRS can be further advanced using larger GWAS, the estimated risk of CHD in individuals at high genetic risk may be altered. Fifth, although the accuracy between self-report and medical record was substantial in life-threatening conditions such as myocardial infraction [39], self-reported information may have recall bias. Therefore, further study using medical records is needed. Nevertheless, this study is meaningful in that it is the first attempt to examine CHD prediction using KRS and GRS in a Korean population. Further studies are expected to investigate the reliability and validity of GRS models using long-term follow-up data. Additionally, in order to prevent CHD and to detect individuals at high risk for CHD early, it is necessary to identify the influence of genetic predisposition according to sex and age and to conduct repeated studies in multiancestry populations.

## 5. Conclusions

Family history and GRS constructed with 55 SNPs were associated with the risk of CHD after adjusting TRFs. Although GRS and family history improved CHD risk identification and reclassification over TRFs, these results did not demonstrate the clinical utility of GRS as a complement to existing CHD risk-assessment tools. For more accurate CHD risk prediction, further studies on various models using GRS should be performed.

## Data Availability

Raw data are available from the National Biobank of Korea (https://nih.go.kr).

## Supplementary Material

Supplementary material associated with this article can be found, in the online version, at https://doi.org/10.31083/j.rcm2404102.

## References

[1] Roth GA, Mensah GA, Johnson CO, Addolorato G, Ammirati E, Baddour LM (2020). Global Burden of Cardiovascular Diseases and Risk Factors, 1990-2019: Update From the GBD 2019 Study. *Journal of the American College of Cardiology*.

[2] Wilson PW, D’Agostino RB, Levy D, Belanger AM, Silbershatz H, Kannel WB (1998). Prediction of coronary heart disease using risk factor categories. *Circulation*.

[3] Goff DC, Lloyd-Jones DM, Bennett G, Coady S, D’Agostino RB, Gibbons R (2014). 2013 ACC/AHA guideline on the assessment of cardiovascular risk: a report of the American College of Cardiology/American Heart Association Task Force on Practice Guidelines. *Journal of the American College of Cardiology*.

[4] Conroy RM, Pyörälä K, Fitzgerald AP, Sans S, Menotti A, De Backer G (2003). Estimation of ten-year risk of fatal cardiovascular disease in Europe: the SCORE project. *European Heart Journal*.

[5] Hippisley-Cox J, Coupland C, Vinogradova Y, Robson J, Brindle P (2008). Performance of the QRISK cardiovascular risk prediction algorithm in an independent UK sample of patients from general practice: a validation study. *Heart*.

[6] Jung KJ, Jang Y, Oh DJ, Oh BH, Lee SH, Park SW (2015). The ACC/AHA 2013 pooled cohort equations compared to a Korean Risk Prediction Model for atherosclerotic cardiovascular disease. *Atherosclerosis*.

[7] Bae JH, Moon MK, Oh S, Koo BK, Cho NH, Lee MK (2020). Validation of Risk Prediction Models for Atherosclerotic Cardiovascular Disease in a Prospective Korean Community-Based Cohort. *Diabetes & Metabolism Journal*.

[8] Ahn KA, Yun JE, Cho ER, Nam CM, Jang Y, Jee SH (2006). Framingham Equation Model Overestimates Risk of Ischemic Heart Disease in Korean Men and Women. *Korean Journal of Epidemiology*.

[9] Jee SH, Jang Y, Oh DJ, Oh BH, Lee SH, Park SW (2014). A coronary heart disease prediction model: the Korean Heart Study. *BMJ Open*.

[10] Khera AV, Kathiresan S (2017). Genetics of coronary artery disease: discovery, biology and clinical translation. *Nature Reviews. Genetics*.

[11] Hughes MF, Saarela O, Stritzke J, Kee F, Silander K, Klopp N (2012). Genetic markers enhance coronary risk prediction in men: the MORGAM prospective cohorts. *PloS one*.

[12] Inouye M, Abraham G, Nelson CP, Wood AM, Sweeting MJ, Dudbridge F (2018). Genomic Risk Prediction of Coronary Artery Disease in 480,000 Adults: Implications for Primary Prevention. *Journal of the American College of Cardiology*.

[13] Morrison AC, Bare LA, Chambless LE, Ellis SG, Malloy M, Kane JP (2007). Prediction of coronary heart disease risk using a genetic risk score: the Atherosclerosis Risk in Communities Study. *American Journal of Epidemiology*.

[14] Ripatti S, Tikkanen E, Orho-Melander M, Havulinna AS, Silander K, Sharma A (2010). A multilocus genetic risk score for coronary heart disease: case-control and prospective cohort analyses. *Lancet*.

[15] Ramírez J, van Duijvenboden S, Young WJ, Tinker A, Lambiase PD, Orini M (2022). Prediction of Coronary Artery Disease and Major Adverse Cardiovascular Events Using Clinical and Genetic Risk Scores for Cardiovascular Risk Factors. *Circulation: Genomic and Precision Medicine*.

[16] Riveros-Mckay F, Weale ME, Moore R, Selzam S, Krapohl E, Sivley RM (2021). Integrated Polygenic Tool Substantially Enhances Coronary Artery Disease Prediction. *Circulation: Genomic and Precision Medicine*.

[17] Mosley JD, Gupta DK, Tan J, Yao J, Wells QS, Shaffer CM (2020). Predictive Accuracy of a Polygenic Risk Score Compared With a Clinical Risk Score for Incident Coronary Heart Disease. *The Journal of the American Medical Association*.

[18] Isgut M, Sun J, Quyyumi AA, Gibson G (2021). Highly elevated polygenic risk scores are better predictors of myocardial infarction risk early in life than later. *Genome Medicine*.

[19] Elliott J, Bodinier B, Bond TA, Chadeau-Hyam M, Evangelou E, Moons KGM (2020). Predictive Accuracy of a Polygenic Risk Score-Enhanced Prediction Model vs a Clinical Risk Score for Coronary Artery Disease. *The Journal of the American Medical Association*.

[20] Semaev S, Shakhtshneider E (2020). Genetic Risk Score for Coronary Heart Disease: Review. *Journal of Personalized Medicine*.

[21] Health Examinees Study Group (2015). The Health Examinees (HEXA) study: rationale, study design and baseline characteristics. *Asian Pacific Journal of Cancer Prevention*.

[22] Kim Y, Han BG, KoGES group (2017). Cohort Profile: The Korean Genome and Epidemiology Study (KoGES) Consortium. *International Journal of Epidemiology*.

[23] Kim HC, Ihm SH, Kim GH, Kim JH, Kim KI, Lee HY (2019). 2018 Korean Society of Hypertension guidelines for the management of hypertension: part I-epidemiology of hypertension. *Clinical Hypertension*.

[24] Hur KY, Moon MK, Park JS, Kim SK, Lee SH, Yun JS (2021). 2021 Clinical Practice Guidelines for Diabetes Mellitus of the Korean Diabetes Association. *Diabetes & Metabolism Journal*.

[25] Moon S, Kim YJ, Han S, Hwang MY, Shin DM, Park MY (2019). The Korea Biobank Array: Design and Identification of Coding Variants Associated with Blood Biochemical Traits. *Scientific Reports*.

[26] Howie BN, Donnelly P, Marchini J (2009). A flexible and accurate genotype imputation method for the next generation of genome-wide association studies. *PLoS Genetics*.

[27] Ishigaki K, Akiyama M, Kanai M, Takahashi A, Kawakami E, Sugishita H (2020). Large-scale genome-wide association study in a Japanese population identifies novel susceptibility loci across different diseases. *Nature Genetics*.

[28] Grambsch PM, Therneau TM (1994). Proportional hazards tests and diagnostics based on weighted residuals. *Biometrika*.

[29] Lu X, Huang J, Wang L, Chen S, Yang X, Li J (2015). Genetic predisposition to higher blood pressure increases risk of incident hypertension and cardiovascular diseases in Chinese. *Hypertension*.

[30] Pencina MJ, D’Agostino RB, Steyerberg EW (2011). Extensions of net reclassification improvement calculations to measure usefulness of new biomarkers. *Statistics in Medicine*.

[31] Pencina MJ, D’Agostino RB, D’Agostino RB, Vasan RS (2008). Evaluating the added predictive ability of a new marker: from area under the ROC curve to reclassification and beyond. *Statistics in Medicine*.

[32] Hajek C, Guo X, Yao J, Hai Y, Johnson WC, Frazier-Wood AC (2018). Coronary Heart Disease Genetic Risk Score Predicts Cardiovascular Disease Risk in Men, Not Women. *Circulation. Genomic and Precision Medicine*.

[33] Pechlivanis S, Lehmann N, Hoffmann P, Nöthen MM, Jöckel KH, Erbel R (2020). Risk prediction for coronary heart disease by a genetic risk score - results from the Heinz Nixdorf Recall study. *BMC Medical Genetics*.

[34] Winham SJ, de Andrade M, Miller VM (2015). Genetics of cardiovascular disease: Importance of sex and ethnicity. *Atherosclerosis*.

[35] Zhao D, Guallar E, Ouyang P, Subramanya V, Vaidya D, Ndumele CE (2018). Endogenous Sex Hormones and Incident Cardiovascular Disease in Post-Menopausal Women. *Journal of the American College of Cardiology*.

[36] Yun H, Noh NI, Lee EY (2022). Genetic risk scores used in cardiovascular disease prediction models: a systematic review. *Reviews in Cardiovascular Medicine*.

[37] Hippisley-Cox J, Coupland C, Robson J, Brindle P (2010). Derivation, validation, and evaluation of a new QRISK model to estimate lifetime risk of cardiovascular disease: cohort study using QResearch database. *British Medical Journal*.

[38] Tada H, Melander O, Louie JZ, Catanese JJ, Rowland CM, Devlin JJ (2016). Risk prediction by genetic risk scores for coronary heart disease is independent of self-reported family history. *European Heart Journal*.

[39] Ho PJ, Tan CS, Shawon SR, Eriksson M, Lim LY, Miao H (2019). Comparison of self-reported and register-based hospital medical data on comorbidities in women. *Scientific Reports*.

