# Supplementary figures and images for: Genetic Risk Score for Prediction of Coronary Heart Disease in the Korean Genome and Epidemiology Study
Source: Rev Cardiovasc Med. 2023 Apr 4;24(4):102. doi: 10.31083/j.rcm2404102 (PMC11273040; doi:10.31083/j.rcm2404102)

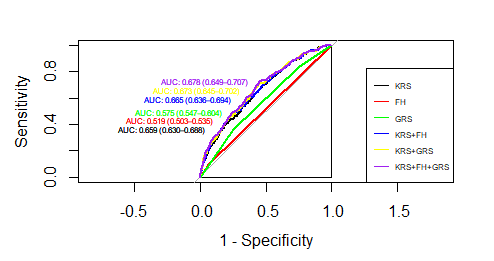

Supplement: Supplementary file 1 [file 2153-8174-24-4-102-s1.zip › Supplementary Fig. 1.png]

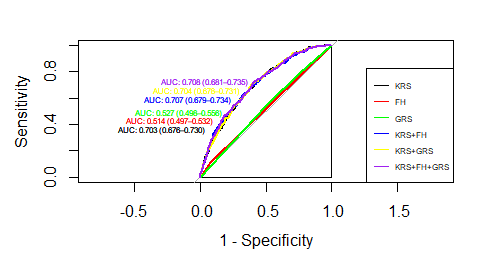

Supplement: Supplementary file 1 [file 2153-8174-24-4-102-s1.zip › Supplementary Fig. 2.png]
